# Phase 1 Dose-Escalation Study of Plasma Kallikrein Inhibitor THR-149 for the Treatment of Diabetic Macular Edema

**DOI:** 10.1167/tvst.10.14.28

**Published:** 2021-12-23

**Authors:** Pravin U. Dugel, Arshad M. Khanani, Brian B. Berger, Sunil Patel, Mitchell Fineman, Glenn J. Jaffe, Petra Kozma-Wiebe, Jeffrey Heier

**Affiliations:** 1 Retinal Consultants of Arizona, Phoenix, AZ, USA; 2 USC Roski Eye Institute, Keck School of Medicine, University of Southern California, Los Angeles, CA, USA; 3 Phoenix Eye Institute, Banner University Medical Center, Phoenix, AZ, USA; 4 Sierra Eye Associates, Reno, NV, USA; 5 The University of Nevada, Reno School of Medicine, Reno, NV, USA; 6 Retina Research Center, Austin, TX, USA; 7 Retina Research Institute of Texas, Abilene, TX, USA; 8 Mid Atlantic Retina, Retina Service of Wills Eye Hospital, Philadelphia, PA, USA; 9 Department of Ophthalmology, Duke University, Durham, NC, USA; 10 Oxurion NV, Leuven, Belgium; 11 Ophthalmic Consultants of Boston, Boston, MA, USA

**Keywords:** diabetic macular edema, kallikrein inhibitor, dose-escalation study

## Abstract

**Purpose:**

The purpose of this study was to evaluate the safety and preliminary efficacy of a single intravitreal injection of 3 dose levels of THR-149 in adults with center-involved diabetic macular edema (DME).

**Methods:**

A phase 1, open-label, multicenter 3 + 3 dose-esclation study with 3-month follow-up. The primary endpoint was the incidence of dose-limiting toxicities (DLTs) up to and including the Day 14 visit. Additional key endpoints included the incidence of (serious) adverse events ([S]AEs), mean change from baseline in best-corrected visual acuity (BCVA) and central subfield thickness (CST), and additional imaging parameters on widefield fluorescein angiography and optical coherence tomography (OCT) angiography.

**Results:**

Twelve subjects were treated: 3 subjects received THR-149 0.005 mg, 3 received 0.022 mg and 6 received 0.13 mg. Baseline ocular characteristics were balanced between subjects at each dose level. There were no DLTs or ocular SAEs, and all subjects completed the study. Six subjects experienced a total of 10 AEs in the study eye; 1 case of mild anterior chamber inflammation was deemed related to THR-149 and/or the injection procedure. Mean change from Baseline in BCVA was +7.5 Early Treatment of Diabetic Retinopathy Study (ETDRS) letters on Day 14, and +6.4 ETDRS letters by Month 3. CST was variable, and mean CST change from baseline was +30.0 µm at Month 3. There were no clinically meaningful changes in imaging parameters.

**Conclusions:**

THR-149 was safe and well tolerated; preliminary efficacy in terms of BCVA improvement was observed.

**Translational Relevance:**

This work bridges the gap between basic research and clinical care by providing first in human safety and preliminary efficacy data, supporting the further investigation of THR-149 as a potential treatment for DME.

## Introduction

The prevalence of diabetic macular edema (DME) is increasing, and this trend is expected to continue in the coming years.^1^^,^^2^ DME is associated with a considerable burden to the healthcare economy and to patients’ quality of life during their working years.^3^^,^^4^ DME is characterized by breakdown of the blood-retinal barrier, leading to vascular leakage and retinal thickening; symptoms of metamorphopsia and vision loss become evident once thickening approaches or involves the fovea.^5^ The pathogenesis of DME is multifactorial and not yet fully understood. Vascular endothelial growth factor (VEGF) is a key factor in the disruption of the blood-retinal barrier,^6^ and anti-VEGF therapies are the standard of care for DME.^7^^,^^8^ However, 20 to 40% of patients do not respond to treatment,^9^^–^^11^ suggesting the involvement of additional pathways in this condition. In addition to VEGF, inflammation plays a key role in the pathogenesis of DME and corticosteroid treatments have shown some efficacy in treating the disease.^12^^,^^13^ However, corticosteroids are associated with cataract formation and elevated intraocular pressure.^12^^–^^14^ Alternative therapeutic targets and treatment strategies are therefore needed.

**Figure 1.**
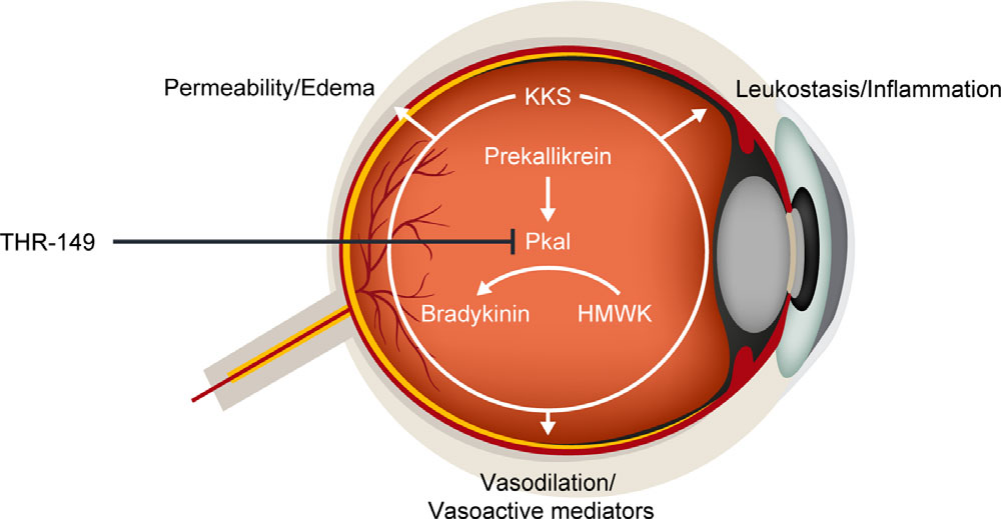
THR-149 is a potent reversible peptide inhibitor of the plasma kallikrein pathway, which mediates vasodilation, permeability, and inflammation. HMWK = high molecular weight kininogen; KKS = kallikrein-kinin system; PKal = plasma kallikrein.

The kallikrein-kinin system contributes to inflammation and vascular dysfunction in animal models of diabetic retinopathy (DR).^15^ Plasma kallikrein (PKal) is a critical mediator of these processes. PKal cleaves kininogen; this action leads to the release of bradykinin, a nonapeptide whose downstream signaling targets promote inflammation, vasodilation, and vascular permeability (Fig. 1). Importantly, these effects are independent of VEGF.^15^^–^^17^ PKal is upregulated in the vitreous of patients with DME, and is not correlated with VEGF expression,^16^ suggesting PKal as a candidate therapeutic target in patients with low VEGF expression, or as a combination therapy with VEGF inhibitors in patients with high expression of both PKal and VEGF. The kallikrein-kinin system is implicated in other conditions that involve edema, and PKal inhibition has demonstrated efficacy as a prophylactic treatment for hereditary angioedema.^18^^,^^19^

THR-149 is a novel, potent inhibitor of human PKal with a molecular weight of approximately 1.7 kilodalton. The molecule structure includes a bicyclic peptide scaffold, characterized by high target affinity and specificity, metabolic stability, and enhanced tissue penetration relative to small molecules or antibodies.^20^^,^^21^ In preclinical studies, THR-149 effectively blocked the release of bradykinin in the vitreous, and reduced the level of edema and retinal vascular leakage in rodent models.^21^ Here, we present safety data and visual and anatomic outcomes from the phase I study of THR-149 in subjects with center-involved DME.

## Methods

The study (ClinicalTrials.gov identifier NCT03511898) was conducted in compliance with the Declaration of Helsinki, the International Council for Harmonisation guideline for Good Clinical Practice, and all applicable local regulatory requirements. The protocol was approved by institutional review boards of all participating sites, and subjects provided written informed consent prior to screening.

### Study Design and Treatment

This multicenter, open-label, phase I study used a single-dose escalation, 3 + 3 design. Eight study visits were scheduled per subject for screening, study treatment, and post-treatment follow up on Day 1, Day 7, Day 14, Month 1, Month 2, and Month 3. A safety monitoring committee made the dose-escalation decisions based on the review of overall safety and dose-limiting toxicities (DLTs) reported by the investigators. Three dose levels of THR-149 were assessed: 0.005 mg, 0.022 mg, and 0.13 mg. At each dose level, 3 subjects received a single intravitreal (IVT) injection of 50 µL solution containing THR-149. Subjects were injected sequentially: subjects 1 and 2 were injected at least 7 days apart, and subjects 2 and 3 were injected at least 2 days apart.

If no DLTs were observed after injection of either of the first two dose levels, then escalation to the third dose level occurred. If no subject developed a DLT after injection of the third and highest dose level, three additional subjects were treated at this dose. At all dose levels, if 1 subject developed a DLT, then 3 additional subjects were treated at the same dose, injected sequentially ≥2 days apart. If none of the additional subjects developed a DLT, then escalation to the next dose level occurred (unless already at the highest dose); otherwise, de-escalation to the previous dose level occurred.

### Subjects

Enrolled subjects were aged ≥18 years of age with center-involved DME and a history of response to prior anti-VEGF and/or corticosteroid treatment in the study eye and in the opinion of the investigator, remained responsive to treatment. Subjects were required to have central subfield thickness (CST) of ≥320 µm on Spectralis spectral domain optical coherence tomography (SD-OCT; Heidelberg Engineering, Heidelberg, Germany), and best-corrected visual acuity (BCVA) Early Treatment Diabetic Retinopathy Study (ETDRS) letter score of ≤62 (i.e. Snellen equivalent 20/63 or worse) and ≥23 (i.e. Snellen equivalent 20/320 or better) in the study eye.

Subjects with macular edema due to causes other than DME (e.g. cataract extraction, vitreomacular traction, and epiretinal membrane), or other potentially confounding concurrent diseases in the study eye, were excluded from the study. Additional exclusion criteria were: presence of neovascularization at the disc, aphakia, uncontrolled glaucoma, or more than 8D high myopia in the study eye; uncontrolled diabetes; and uncontrolled hypertension. Subjects with prior confounding treatments or procedures, such as anti-VEGF therapy within 1 month, intraocular surgery or focal/grid laser within 3 months, or panretinal laser photocoagulation or dexamethasone IVT implant within 6 months, were also excluded.

### Endpoints and Assessments

The primary endpoint was the incidence of DLTs up to and including the Day 14 visit. A DLT was defined as intraocular inflammation of ≥2+ on any of the intraocular inflammation grading scales (grading of anterior chamber cells, grading of anterior chamber flare, and Nussenblatt chart for vitreous inflammation grading)^22^^,^^23^ or a decrease in BCVA of ≥10 ETDRS letters from baseline.

Secondary endpoints were incidence of systemic and ocular (serious) adverse events ([S]AEs) until study end, and occurrence of hematological, biochemical, or urinary laboratory abnormalities by study visit. Exploratory endpoints included change from baseline in BCVA and CST (based on SD-OCT) by study visit and PKal levels in plasma samples by Day 7. Exploratory imaging outcomes included ischemia and leakage assessments by Month 3 based on widefield fluorescein angiography, vascular density, and foveal avascular zone area by study visit based on OCT angiography (only at sites with the required equipment).

At each visit, the investigator carried out a full ophthalmic examination (slit lamp and dilated fundus examinations, and assessment of intraocular pressure), as well as BCVA and SD-OCT assessments (SD-OCT was assessed at all visits except the injection visit). Fluorescein angiography and color fundus photography were performed at Screening and at Month 3, whereas OCT angiography was performed at Screening, Month 1 and Month 3. A central reading center assessed all ophthalmic imaging parameters (Duke Reading Center, Durham, NC, USA). Further details on imaging acquisition are included in the Supplementary Methods (available at www.ophthalmologyretina.org).

### Statistical Methodology

The all treated subjects analysis set was used for all analyses in the study and included all subjects who received an injection of THR-149. Data were analyzed by dose level, using descriptive statistics. Analysis of BCVA and SD-OCT parameters were adjusted to account for rescue treatment during the follow-up period, with the last value at rescue carried forward. Rescue treatment was defined as any standard of care treatment for DME (i.e. anti-VEGF agent or corticosteroid) given intravitreally in the study eye during the 3-month follow-up period. Missing data were not imputed. Descriptive summary statistics for continuous variables included mean, standard deviation, standard error, median, and range. Categorical data were summarized by count and percentage. Ninety-five percent confidence intervals for proportions were calculated using the Exact Binomial Clopper-Pearson method.

## Results

Twelve subjects were enrolled at five sites in the United States. All 12 subjects received a single IVT injection of THR-149; 3 received 0.005 mg (referred to hereafter as “low dose”), 3 received 0.022 mg (“middle dose”), and 6 received 0.13 mg (“high dose”). All subjects attended all scheduled visits and completed the study. Demographics and baseline clinical characteristics are shown in Table 1. Baseline ocular characteristics were balanced between subjects treated at each dose. All subjects had moderate nonproliferative DR in the study eye as assessed by the central reading center at baseline and received their last anti-VEGF injection at least 1 month prior to study treatment. The mean (SD) baseline BCVA was 44.7 (10.19) ETDRS letters and mean (SD) baseline CST was 524.0 (89.49) µm.

**Table 1.** Demographics and Baseline Clinical Characteristics

	THR-149 Dose Level			
Characteristic	Low (*N* = 3)	Middle (*N* = 3)	High (*N* = 6)	Overall (*N* = 12)
Age (y)				
Mean (SD)	65.0 (7.21)	65.3 (8.14)	72.2 (10.98)	68.7 (9.47)
Median	67.0	69.0	76.5	70.5
Range	57–71	56–71	51–81	51–81
Gender, *n* (%)				
Male	0	2	4	6 (50.0)
Female	3	1	2	6 (50.0)
Race, *n* (%)				
White	3	3	5	11 (91.7)
Black/African American	0	0	1	1 (8.3)
BCVA (ETDRS letters)				
Mean (SD)	46.0 (9.17)	46.7 (8.62)	43.0 (12.59)	44.7 (10.19)
Median	44.0	45.0	43.5	44.5
Range	38–56	39–56	25–58	25–58
CST (µm)				
Mean (SD)	497.7 (70.04)	539.3 (35.95)	529.5 (120.60)	524.0 (89.49)
Median	533.0	551.0	585.0	547.0
Range	417–543	499–568	373–626	373–626
Prior treatment for DME, *n* (%)				
Anti-VEGF	3	3	6	12 (100.0)
Corticosteroids	1	1	4	6 (50.0)
Prior ocular interventions, *n* (%)				
Cataract surgery	1	3	5	9 (75.0)
Focal/grid laser^a^	1	1	1	3 (25.0)
Panretinal photocoagulation^a^	0	0	2	2 (16.7)
Epiretinal membrane,^b^ n (%)	3	2	5	10 (83.3)
Subretinal fluid, *n* (%)	2	0	0	2 (16.7)

BCVA = best-corrected visual acuity; CST = central subfield thickness; DME = diabetic macular edema; ETDRS = Early Treatment Diabetic Retinopathy Study; SD = standard deviation; VEGF = vascular endothelial growth factor.

a Intervention was >6 months prior to study treatment and therefore did not require exclusion.

b Epiretinal membrane was not the cause of macular edema and therefore did not require exclusion.

Prior to Month 3, 3 subjects received ≥1 rescue treatment. At the low-dose and middle-dose levels, 1 subject each received a first rescue treatment at Month 1. At the high-dose level, 1 subject received a first rescue treatment at month 2. Rescue treatment was aflibercept (2 mg in 0.05 mL) in all instances.

### Safety Analysis

No DLTs or ocular SAEs were reported at any dose level. Overall, 6 subjects (50%) experienced a total of 10 AEs in the study eye (Table 2). All were mild to moderate in severity, most occurred at the middle-dose level, and 5 of the 10 events occurred within 7 days of study treatment. One AE in the study eye was deemed treatment-related by the investigator, meaning that it could be related to either the drug or the injection procedure. This was a case of anterior chamber inflammation affecting a subject at the middle-dose level that started on Day 1, was mild in severity, resolved by Day 5, and did not require treatment.

**Table 2.** Summary of Adverse Events

	THR-149 Dose Level							
	Low (*N* = 3)		Middle (*N* = 3)		High (*N* = 6)		Overall (*N* = 12)	
Characteristic	*n*	E	*n*	E	*n*	E	*n*, %	E
DLTs	0	0	0	0	0	0	0	0
SAE	0	0	1	3^a^	0	0	1 (8.3)	3
AEs in the study eye								
Anterior chamberinflammation	0	0	1^b^	1	0	0	1 (8.3)	1
Conjunctivalhemorrhage	0	0	1	1	0	0	1 (8.3)	1
Corneal disorder	0	0	1^c^	1	0	0	1 (8.3)	1
Diabetic retinaledema	1	1	1	1	1	1	3 (25.0)	3
Eye pain	0	0	0	0	1	1	1 (8.3)	1
Macular fibrosis^d^	0	0	1	2	0	0	1 (8.3)	2
Vitreous floaters	1	1	0	0	0	0	1 (8.3)	1

AE = adverse event; DLT = dose-limiting toxicity; E = number of events; n = number of subjects with an event; SAE = serious adverse event.

All events occurred within 7 days postinjection, except for diabetic retinal edema and macular fibrosis.

a Hemorrhagic anemia (2 events), and pneumonia; none deemed treatment-related (drug and/or procedure) by the investigator.

b Deemed related to study treatment and/or injection procedure by the investigator; occurred on day 1, was mild in severity, and resolved by day 5.

c Corneal folds; not deemed related to study treatment and/or injection procedure by the investigator, occurred on day 7, was mild in severity, and resolved by day 12.

d Epiretinal membrane with deformation in the center 1 mm; 2 occurrences in a single subject, both of which were not deemed related to study treatment and/or injection procedure by the investigator and were mild in severity. The first occurrence started on day 14 and resolved by day 18, whereas the second occurrence started at the last study visit (month 3) and resolved a little over 1 month later (day 126).

Three nonocular SAEs (2 events of hemorrhagic anemia and 1 of pneumonia) were reported in 1 subject at the middle-dose level, with onset after Month 1; none were deemed to be treatment-related, and all were moderate in severity and resolved during follow-up. The same subject experienced visual acuity loss of 6 ETDRS letters from baseline at Month 1, which returned to baseline by Month 2 and remained stable until study end. No other subjects experienced visual acuity loss of ≥5 letters during the study. Fluctuations in intraocular pressure were as expected for an IVT injection. Mean change in intraocular pressure from pre- to post-injection on the day of treatment was +5.7 mm Hg, −3.7 mm Hg, and +1.7 mm Hg at the low-, middle-, and high-dose levels, respectively; values returned to baseline at all dose levels by the Day 1 follow-up visit. THR-149 was undetectable in the plasma samples of all subjects at all time points measured (baseline, day 1, and day 7; lower limit of quantification = 1 ng/mL). Any abnormal laboratory values were in line with underlying disease, and there were no signs of any significant laboratory abnormalities that developed following the IVT injection with THR-149.

### Visual and Anatomic Outcomes

**Figure 2.**
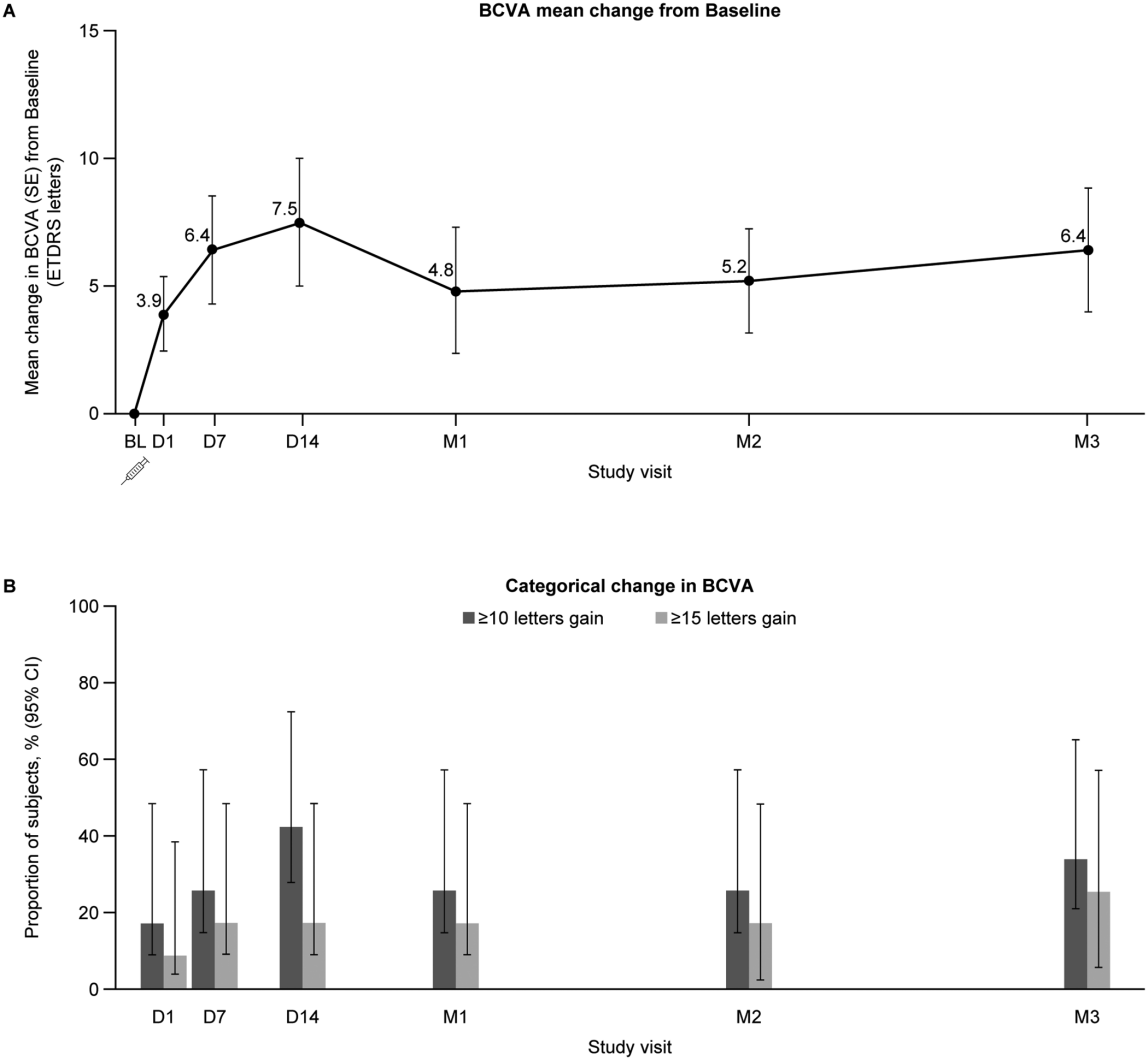
Overall mean change from baseline in BCVA over time (**A**), and BCVA gain ≥10 letters or ≥15 letters (**B**). For subjects who received rescue treatment (2 subjects at month 1 and 1 subject at month 2), the value before rescue was carried forward. BCVA = best-corrected visual acuity; BL = baseline; CI, confidence interval; ETDRS = Early Treatment Diabetic Retinopathy Society; D = day; M = month; SE = standard error.

Mean BCVA increased from Day 1 post-treatment; increases were noted at all dose levels at all study visits and remained above baseline until Month 3 (Fig. 2A). Mean BCVA increase from baseline was highest at Day 14, at 7.5 ETDRS letters (14.0, 8.7, and 3.7 letters at the low-, middle-, and high-dose levels, respectively), and was 6.4 ETDRS letters above baseline at Month 3 (9.0, 7.0, and 4.8 letters at the low-, middle-, and high-dose levels, respectively). At Day 14, 5 of 12 subjects (42%) had a BCVA gain of ≥10 ETDRS letters from baseline, including 2 subjects who gained ≥ 15 letters. At Month 3, 4 of 12 subjects (33%) had a BCVA gain of ≥ 10 letters, including 3 with gains of ≥ 15 letters (Fig. 2B).

**Figure 3.**
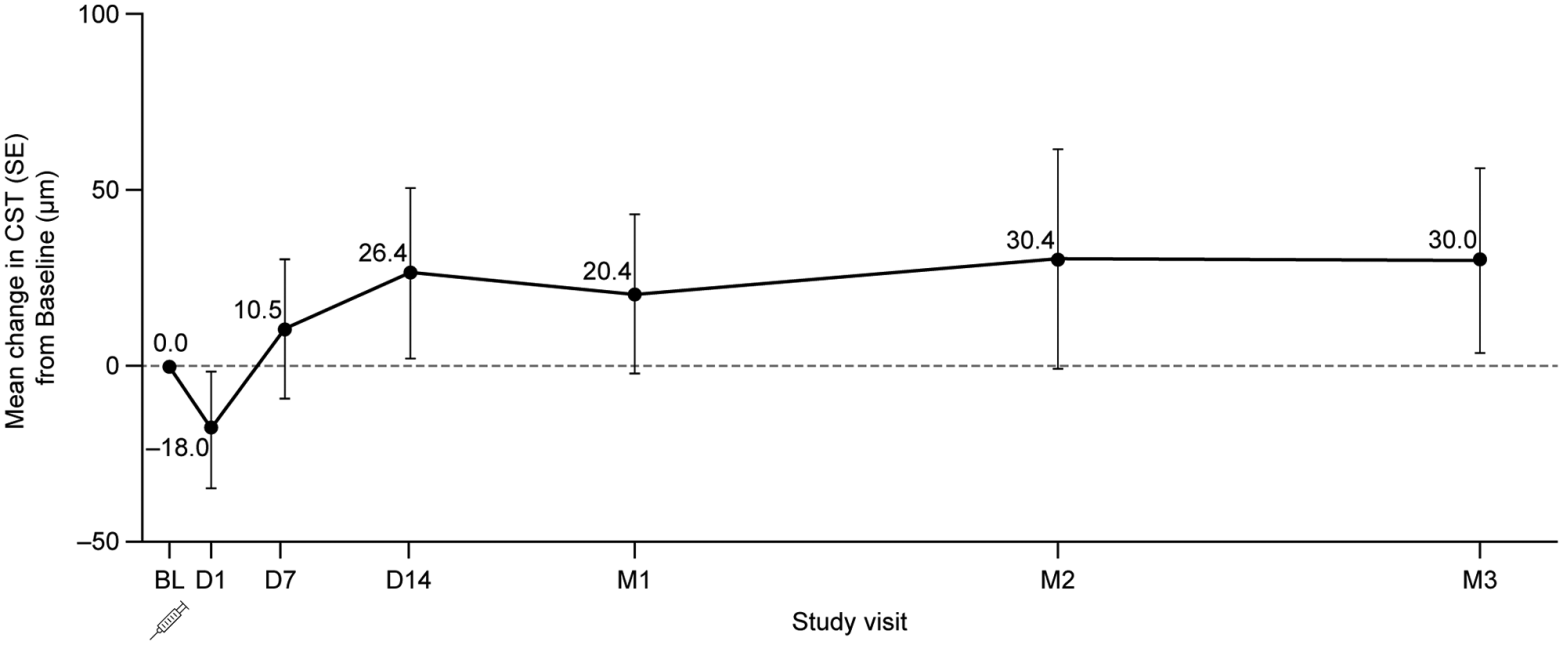
Mean change from baseline in CST over time. For subjects who received rescue treatment, value before rescue was carried forward. BL = baseline; CST = central subfield thickness; D = day; M = month; SE = standard error.

A marginal decrease in CST was noted at Day 1, with an overall mean decrease from baseline of 18.0 µm. This was followed by an increase that was maintained to Month 3 (Fig. 3). The largest observed mean change from baseline in CST was an increase of 30.4 µm at Month 2, although variability was high. A clear dose-response relationship was not evident; subjects treated at the low-dose level appeared to have the best improvement in BCVA, whereas those treated at the high-dose level had a more favorable CST profile (Supplementary Figure, available at www.ophthalmologyretina.org).

No persistent clinically meaningful changes from baseline were observed for any of the SD-OCT parameters. Two subjects without subretinal fluid (SRF) at baseline had SRF at Month 1 and Month 3, respectively, and one subject with SRF at baseline had no SRF at Month 2. On OCT angiography, performed in only 4 subjects, there were no persistent clinically meaningful changes from baseline to Month 3.

Fluorescein angiography parameters including mean nonperfusion index, neovascularization elsewhere, vascular leakage index, and foveal avascular zone area were similar at Baseline and Month 3 for all subjects. From Baseline to Month 3, macular capillary leakage developed in 1 subject at the low-dose level and resolved in 1 subject (without rescue treatment) at the high-dose level. There was no change from baseline in severity of DR for any subject during the study, as assessed by color fundus photography.

## Discussion

In this phase I study, we show that THR-149 was safe and well tolerated in subjects with center-involved DME who had received prior anti-VEGF therapy. There were no DLTs or serious ocular AEs. One case of anterior chamber inflammation was reported as treatment-related, meaning it could be attributed to either the treatment or the injection procedure. Intraocular inflammation is associated with IVT injection procedures irrespective of the therapy administered^24^^,^^25^ and this single, mild case resolved without treatment. Overall, no safety signals of concern were associated with the study treatment. These observations were consistent with preclinical findings, which showed no overt toxicological effects, even at the maximum tested dose.^21^

THR-149 was undetectable in the plasma samples of all subjects at all time points measured (Baseline, Day 1, and Day 7). This is in line with pharmacokinetic data obtained in cynomolgus monkeys, where THR-149 administered bilaterally at 0.13 mg/eye remained detectable in plasma up to 24 hours after the IVT injections, whereas no measurable amount of THR-149 was detected in plasma after bilateral administration of THR-149 0.05 mg/eye, even at the earliest time point tested (30 minutes after the IVT injection). The low concentration of THR-149 in the systemic circulation following IVT injection is supported by a mathematical model that was developed using pharmacokinetic data obtained in New Zealand White rabbits, which predicts that <2% of the total administered drug is present in the systemic circulation at any point in time. At the same time, an IVT half-life of 36 hours was observed in the rabbit, and the fraction of THR-149 present in the ocular tissues compartement (a compartement through which THR-149 transits when being drained from the vitreous into the systemic circulation) is expected to be the highest around 29 hours postinjection.^26^ THR-149 was engineered to be highly resistant to proteolytic degradation, and its stability is supported by preclinical evidence, including the absence of detectable metabolic degradation following IVT administration in the rabbit. It is therefore anticipated that THR-149 will also be highly stable in the human eye.^26^

Mean BCVA increased from Day 1 postinjection, and 5 of 12 subjects gained 10 ETDRS letters or more by Day 14. Overall, mean BCVA remained above baseline levels until Month 3. Mean CST was increased until Month 3, showing an apparent disconnect with BCVA. In general, studies have shown an inverse correlation between CST and BCVA in subjects with DME who receive anti-VEGF or laser treatment, although the correlation tends to be modest and individual patients may experience BCVA improvement without a decrease in CST.^27^^,^^28^ The reason for such a disconnect is unclear, although a recent analysis suggested a better correlation between CST and BCVA in patients who have high baseline CST (>650 µm),^27^ which would not apply to subjects in our study. Delayed anatomic responses to anti-VEGF therapy have also been documented, and early BCVA response is suggested to be a superior indicator of long-term outcome.^29^ Given the VEGF-independent mechanism of action of THR-149, CST may be a less relevant marker of early THR-149 activity. Further investigation in a larger cohort of patients may help to clarify the dynamics of CST and BCVA changes in subjects receiving THR-149.

Rescue treatment was allowed to be administered at the discretion of the investigator when a subject lost ≥10 ETDRS letters from Baseline and / or had an increase of ≥50 µm in CST from Baseline. Three (3) subjects received ≥1 rescue treatment. All three subjects were administered rescue treatment following an increase in CST. In order to assess the effect of THR-149 alone, BCVA and CST values post-rescue treatment were not used in the efficacy analyses. Instead, the last value before rescue treatment was carried forward.

A recent phase I study of another PKal inhibitor, KVD001, enrolled 14 subjects with center-involved DME who had received prior anti-VEGF treatment.^30^ Baseline demographics were similar to those in the current study, with some notable differences in baseline ocular characteristics, including higher baseline BCVA and lower CST relative to those enrolled in our study. The maximum BCVA increase following a single IVT injection of KVD001 was 4.1 ETDRS letters at Month 3, with a corresponding mean reduction of 23 µm in CST. Rescue treatment was allowed; however, the frequency and imputation method were not reported. Comparison of these studies is inevitably confounded by differences in subject characteristics and study methodologies, as well as small sample sizes.

THR-149 is administered intravitreally in order to achieve high, therapeutically effective concentrations in the vitreous, whereas limiting overall systemic exposure and potential systemic side effects. Oral dosing would result in considerably higher systemic exposure and may not lead to pharmacologically active concentrations in the eye. At the same time, oral therapies targeting pKal are currently in development for the treatment of DME (e.g. RZ-402, VE-3539, KV998076 and KV123833). Although most are still in preclinical development, positive topline results from the phase Ia study with RZ-402 have recently been announced. The study enrolled 30 healthy adult volunteers in 3 planned sequential dose-level cohorts of 25 mg, 100 mg and 250 mg. Within each 10-subject dose cohort, subjects were randomized 8:2 to receive either RZ-402 or placebo. A single oral administration of RZ-402 was generally safe and well-tolerated and resulted in plasma concentrations that substantially exceeded target pharmacologically active drug levels (3.5 ng/mL; the concentration that was pharmacologically active in animal models of DME for a 24-hour period; Rezolute press release, May 4, 2021).

In general, these data highlight the expansion of the therapeutic landscape beyond VEGF inhibition for the treatment of DME, and the need for further investigation of these agents in larger studies.

A strength of the current study was the use of a CRC for ophthalmic imaging. Visual outcome analyses can be considered conservative due to imputation of the last value before rescue treatment; however, interpretation is inevitably limited by the small sample size. Overall, preliminary safety and visual acuity outcomes are encouraging, and further investigation is justified. To this end, a phase II study will evaluate THR-149 in a larger cohort of subjects with DME.

In conclusion, this phase I study shows that a single IVT injection of THR-149 was safe and well-tolerated. Preliminary data suggest activity in subjects with DME, with immediate gains in BCVA that were maintained throughout the duration of the study. These data support further clinical investigation with multiple injections of THR-149. This work bridges the gap between basic research and clinical care by providing first in human safety data and providing preliminary efficacy in terms of BCVA increase.

## Supplementary Material

Supplement 1

Supplement 2
