# Supplementary material for: Phase 1 Dose-Escalation Study of Plasma Kallikrein Inhibitor THR-149 for the Treatment of Diabetic Macular Edema
Source: Transl Vis Sci Technol. 2021 Dec 23;10(14):28. doi: 10.1167/tvst.10.14.28 (PMC8711005; doi:10.1167/tvst.10.14.28)

Supplemental Figure. Mean change in BCVA (A) and CST (B) by dose level.

BCVA = best-corrected visual acuity; BL = Baseline; CST = central subfield thickness; ETDRS = Early Treatment Diabetic Retinopathy Society; D = Day; M = Month; SE = standard error.

A

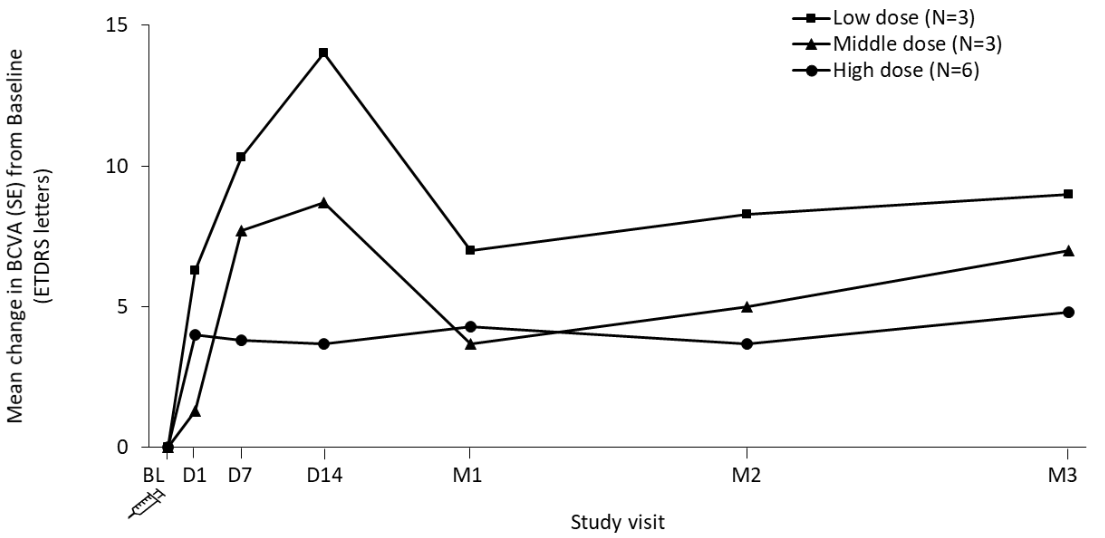

B

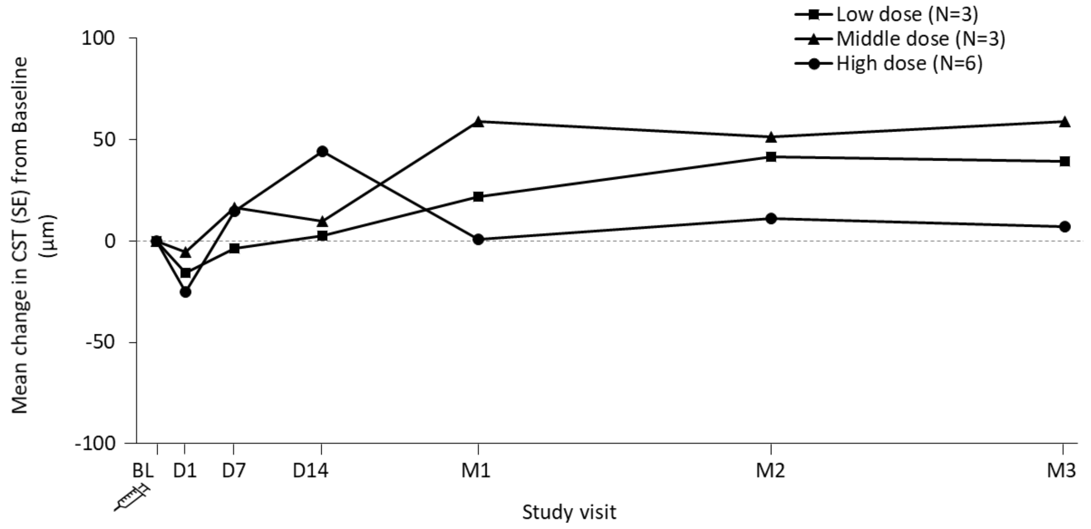

Supplement: Supplement 1 [file tvst-10-14-28_s001.pdf]
