# Supplementary material for: Phase 1 Dose-Escalation Study of Plasma Kallikrein Inhibitor THR-149 for the Treatment of Diabetic Macular Edema
Source: Transl Vis Sci Technol. 2021 Dec 23;10(14):28. doi: 10.1167/tvst.10.14.28 (PMC8711005; doi:10.1167/tvst.10.14.28)
Supplement: Supplement 2 [file tvst-10-14-28_s002.pdf]

Supplemental Methods: Imaging

All imaging scans were submitted to and graded by the central reading center (CRC; Duke Reading Center). All equipment and study personnel taking the scans were certified by the CRC; the certified equipment was used by certified study personnel throughout the entire study.

Spectral domain optical coherence tomography (SD-OCT) was used to assess 3 parameters for retinal thickness: central subfield thickness (CST), retinal thickness in center point and total volume of retinal thickness in the 6-mm Early Treatment of Diabetic Retinopathy Study (ETDRS) grid. Other parameters included vitreomacular traction with deformation in the center 1 mm, epiretinal membrane, intraretinal fluid, subretinal fluid, disorganization of retinal inner layers (DRIL), foveal DRIL, ellipsoid zone integrity in the center 1 mm, external limiting membrane integrity in the center 1 mm, and presence of retinal anomaly.

SD-OCT imaging was done with Heidelberg Spectralis® with software version 5.1 or higher; equipment and study personnel taking the SD-OCT scans were all certified by the CRC (Duke Reading Center).

Widefield fluorescein angiography was carried out with the Fluorescein Angiography Optomap (200° widefield). Parameters assessed were nonperfusion index (percentage of the total area of capillary nonperfusion over the total retinal area), vascular leakage index (percentage of the total area of vascular leakage over the total retinal area), classification of capillary leakage, foveal avascular zone area, neovascularization of the disc, and neovascularization elsewhere.

OCT angiography was used to assess superficial capillary density in the 1-mm and the 3-mm ring, deep capillary plexus density in the 1-mm and the 3-mm ring, foveal avascular zone area and the presence of any retinal vascular anomaly. OCT angiography was done only at sites that had Optovue OCT-Angiography equipment.
